# Metastasis of Breast Lobular Carcinoma to the Uterine Cervix: A Narrative Review

**DOI:** 10.3390/diagnostics16121925

**Published:** 2026-06-21

**Authors:** Mahmoud Rezk Abdelwahed Hussein, Toka Mahmoud Rezk Abdelwahed Hussein

**Affiliations:** 1 Department of Pathology, Faculty of Medicine, Assiut University Hospitals, Assiut 71515, Egypt; 2 Faculty of Medicine, Sohag University, Sohag 82524, Egypt; dereer681@gmail.com

**Keywords:** lobular, carcinoma, cervix, metastasis

## Abstract

**Background:** Metastases to the uterine cervix from extragenital malignancies represent uncommon clinical events, with breast invasive lobular carcinoma (ILC) documented as the predominant primary source in reported literature. **Objectives/Aim:** To characterize the clinicopathologic features of ILCs metastatic to the uterine cervix. **Methods:** We performed a PubMed search using several keywords. **Results:** A total of 29 studies were included in the final analysis. The mean age at presentation of cervical metastasis was 56.8 ± 2.0 years. The mean interval between the initial diagnosis of ILC and the detection of cervical metastasis was 55.6 ± 8.2 months. Clinical presentations included vaginal bleeding, pelvic pain, and unhealthy enlarged, indurated uterine cervix on local examination. The diagnosis was established via tissue biopsy and immunohistochemical stains (positive reactivity for CK7, ER, PR, E-Cadherin, GATA3, GCDP-15 and mammaglobin). There are no consensus treatment protocols, and therapy should be tailored individually based on the extent of disease. Combined surgical and systemic therapy was the most commonly used modality. **Conclusions:** Metastasis of breast ILCs to the uterine cervix poses a significant diagnostic challenge. A high index of clinical suspicion and detailed clinical history are essential for accurate diagnosis.

## 1. Introduction

The female reproductive organs are susceptible to metastatic spread from distant primary cancers, though such occurrences remain rare [1]. Metastases to the uterine corpus from non-gynecological origins are particularly rare [2]. When identified, breast cancer—specifically invasive lobular carcinoma (ILC)—represents a primary source [3]. Historical studies have documented that breast carcinomas account for a significant proportion of metastatic lesions to the uterine cervix, with ILC demonstrating particular predilection for this site [4].

Previous studies indicated that about 30% of breast cancer patients develop metastases even after receiving proper management. Patients with ILCs, but not those with invasive ductal carcinoma (IDC), usually have metastases at the time of diagnosis. Metastasis from IDC usually affects the lungs, brain, bone and liver [5,6]. ILC exhibits a distinctive metastatic distribution pattern. Metastasis from this tumor demonstrates preferential involvement of the female genital tract (endometrium and cervix), peritoneum–retroperitoneum, gastrointestinal tract, bone marrow and adrenal glands [7].

The diagnosis of metastatic ILCs to the uterine cervix is almost always delayed for several reasons. The metastatic disease typically presents with non-specific gynecologic symptoms, namely abnormal and irregular vaginal bleeding. According to the seminal work by Mazur et al. [8], approximately 42% of metastatic tumors involving the uterine cervix are misdiagnosed as primary neoplasms of the uterine cervix, mostly leiomyoma or primary cervical adenocarcinoma [8]. Moreover, metastasis usually occurs after a decade-long disease-free interval, probably due to expression of the estrogen and progesterone receptors by the tumor cells [7]. Metastases from ILCs to the uterine cervix can be discovered synchronously, or prior to or many decades after the diagnosis of the primary ILCs [9].

## 2. Resistance of the Uterine Cervix to Metastatic Spread

### 2.1. General Considerations

**Figure 1:**
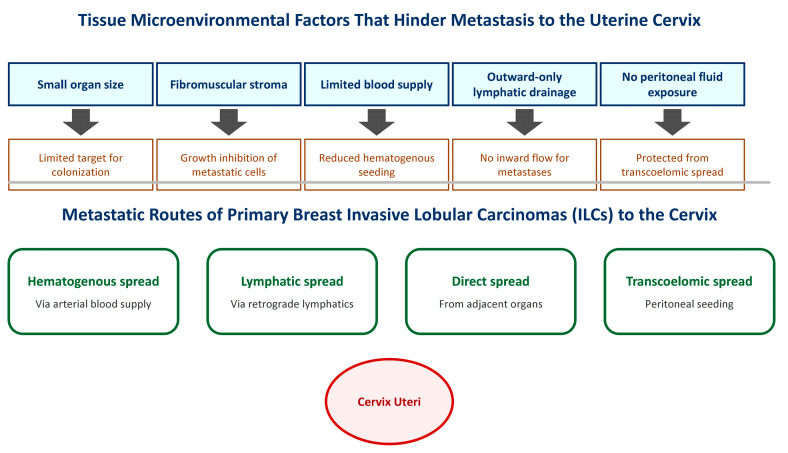
Microenvironmental barriers and clinical outcomes of metastatic spread to the uterine cervix. The schematic illustrates the tissue-specific factors that contribute to the exceptional rarity of cervical metastasis from primary breast ILCs. The upper panel identifies five key inhibitory factors: small organ size, which limits the colonizable target area; fibromuscular stroma, which actively inhibits metastatic cell growth; limited blood supply, reducing the frequency of hematogenous seeding; outward-only lymphatic drainage, preventing retrograde lymphatic spread; and a lack of peritoneal fluid exposure, which protects the organ from transcoelomic (peritoneal) seeding. These factors collectively impede standard metastatic routes, including arterial, retrograde lymphatic, and direct spread. The lower panel highlights the biological progression where these barriers typically lead to failed metastasis, resulting in the extremely rare clinical occurrence of successful cervical colonization by malignant cells from breast ILCs.

The uterine cervix demonstrates remarkable resistance as an anatomical structure to establishing metastatic deposits, whether through lymphatic or vascular routes, from pelvic or extrapelvic primary malignant tumors. This characteristic resistance is well documented in pathology literature and contrasts sharply with common metastatic sites such as the liver, lungs, and bone [10,11]. As originally proposed by Wallach and Edberg [10], cervical metastasis is rare due to the small size of the uterine cervix and its limited blood and lymphatic supply. Moreover, the microenvironment of the uterine cervix (fibromuscular stroma) is not favorable for tumor growth [10]. In particular, the uterine cervix in postmenopausal females is characterized by increased fibrous tissue and reduced blood supply. Taken together, these factors create a microenvironment that is less favorable for the growth of metastatic tumor deposits [12,13]. A summary of the microenvironmental barriers and clinical outcomes of metastatic spread to the uterine cervix is depicted in Figure 1.

### 2.2. Resistance to Vascular Spread

The uterine cervix receives its blood supply from the descending cervical branch of the uterine artery. Its venous drainage occurs through the uterine venous plexus into the internal iliac veins and inferior vena cava [14]. This vascular pattern implies that circulating malignant cells from sites such as the breast, gastrointestinal tract, and lungs must first pass through pulmonary or hepatic capillary beds—the primary filters—before reaching the arterial network supplying the uterine cervix [15]. This architecture markedly reduces the likelihood of hematogenous spread to the uterine cervix.

As a fibromuscular, low-blood-flow “end-organ,” uterine cervix is not a high-volume filtration site. This contrasts with organs such as the liver and lungs, which have high vascular flow and are common sites for metastasis [16]. Additionally, the cervical stroma is relatively insensitive to steroid hormones, making it an unfavorable milieu for hormone-responsive metastases (e.g., breast cancer). It also lacks specific chemokine signaling (e.g., CXCL12) and growth factors that attract and sustain hematogenously disseminated malignant cells [17] (Figure 1).

### 2.3. Resistance to Jymphatic Spread

The uterine cervix has a rich lymphatic outflow network that drains toward pelvic and extrapelvic nodes. In contrast, it has poor lymphatic inflow, making it a poor recipient of metastatic cells via lymphatics [11,12,18]. This is attributed to the absence of primary lymphatic vessels directing lymph from other organs into the fibromuscular stroma of the uterine cervix [19]. Despite these limitations, metastasis can still occur, albeit rarely, through retrograde lymphatic flow. This typically occurs with extensive lymphatic obstruction caused by advanced pelvic malignancy [11]. Histologically, clusters of metastatic cells may obstruct cervical lymphovascular spaces, supporting lymphatic spread from primary malignancies outside the pelvis [18].

### 2.4. Resistance to Transcoelomic Spread

Transcoelomic (peritoneal) spread is typical of ovarian cancer but is rarely encountered in the uterine cervix. This is because the ectocervix is located entirely within the vagina, below the peritoneal reflection. The supravaginal portion of uterine cervix has only limited posterior peritoneal exposure (Douglas pouch). These anatomical features reduce exposure to free-floating malignant cells and therefore make transcoelomic (peritoneal) spread of malignant cells to the uterine cervix an exceptionally rare event [20] (Figure 1).

## 3. Clinical, Immunohistological, and Molecular Features

ILC accounts for approximately 10–15% of all breast cancers and exhibits distinct metastatic patterns compared to IDC [5,6]. Gynecological involvement—particularly of the uterus and uterine cervix—represents an unusual but well-documented metastatic site in ILC.

### 3.1. Clinical Features of Metastasis from ILC to the Uterine Cervix

Cervical metastases from ILC usually occur years after the initial diagnosis, frequently during hormonal therapy or routine follow-up [21]. Abnormal irregular vaginal bleeding, including postcoital bleeding, is a common symptom [22]. However, presentations may be atypical, including abdominal pain or incidental findings (atypical cells in Pap smears) [23,24,25,26]. In 2012, Montiel and colleagues reviewed 30 articles on metastatic breast carcinomas to the uterine cervix, identifying 36 patients. Half of these patients had mammary carcinomas with metastasis to the uterine cervix and demonstrated prolonged survival rates [2].

### 3.2. Immunohistological Features of Metastasis from ILC to the Uterine Cervix

Histologically, metastatic ILCs involving the uterine cervix have characteristic features. These include the lack of glandular dysplasia of the endocervical glandular epithelium or an in situ carcinomatous component and absence of involvement of the surface squamous or glandular epithelium. Other features include deep involvement of the fibromuscular stroma of the uterine cervix and extensive involvement of stromal lymphatics or blood vessels [20]. The infiltrating tumor cells of metastatic ILCs shows infiltration of the stroma by small, banal-looking, uniform cells arranged in a single file (Indian file), solid, or patternless patterns within a dense desmoplastic stroma [27,28]. The differential diagnosis includes primary carcinomas of the gastrointestinal and gynecological tracts [29].

**Figure 2:**
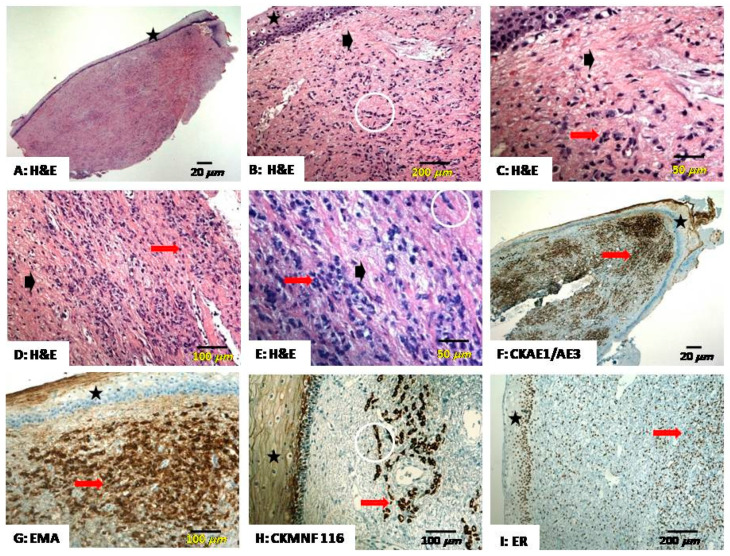
Histopathological and immunohistochemical features of metastatic ILC to the uterine cervix. (**A**,**B**) H&E-stained histological sections of the cervical stroma at low to medium magnification show an infiltrative malignant neoplasm composed of discohesive, small, relatively uniform malignant cells. (**C**) At higher magnification, the cells exhibit the classic “Indian file” linear arrangement characteristic of ILC. (**D**,**E**) H&E: higher magnification further illustrates the bland cytology of the tumor cells, which can easily be mistaken for inflammatory cells, fibrocytes, dendritic cells, or stromal histiocytes. (**F**) CKAE1/AE3: Strong, diffuse cytoplasmic positivity for pancytokeratin confirms the epithelial nature of the infiltrating malignant cells and highlights the extensive stromal involvement. (**G**) Epithelial membrane antigen (EMA) shows strong membranous and cytoplasmic staining, supporting a diagnosis of carcinoma. (**H**) CKMNF116: additional cytokeratin staining confirms the presence of malignant epithelial cells within the fibromuscular stroma of the uterine cervix. (**I**) Strong and diffuse nuclear positivity for estrogen receptor (ER) is highly suggestive of a primary breast origin, particularly in the context of the observed lobular morphology. Magnification: (**A**) ×20, (**B**) ×100, (**C**) ×400, (**D**) ×200, (**E**) ×400, (**F**) ×20, (**G**) ×200, (**H**) ×200, and (**I**) ×100. The black stars indicate the unremarkable covering squamous epithelium of the uterine cervix. The black arrowheads mark the subepithelial cervical stroma. The red arrows point to groups and cords of ILC cells. The white circles indicate the characteristic single-file (Indian file) infiltration pattern of ILC cells invading the cervical stroma. Abbreviations: H&E: hematoxylin and eosin; ILC: invasive lobular carcinoma; CKAE1/AE3: cytokeratin AE1/AE3; EMA: epithelial membrane antigen; CKMNF116: cytokeratin MNF116; ER: estrogen receptor.

Immunohistochemistry is essential for confirmation [30]. Based on established diagnostic criteria in the literature, metastatic ILC typically demonstrates the following immunoprofile: positive reactivity for CK7, GATA3, ER, PR, GCDFP-15, and mammaglobin, with negative staining for CK20, PAX8, and CDX2 [30,31]. A summary of the immunohistological features of metastatic ILCs to the uterine cervix is depicted in Figure 2.

### 3.3. Molecular Features of Metastasis from ILC to the Uterine Cervix

The pathogenesis of ILC metastasis to the uterine cervix remains poorly understood [16]. A hallmark feature of ILC is the loss of E-cadherin protein expression, encoded by the *CDH1* gene, which functions as a cell adhesion molecule [32]. The loss of E-cadherin protein leads to the characteristic non-cohesive growth pattern of malignant cells. This loss may occur through various molecular mechanisms including somatic mutations, loss of heterozygosity, or epigenetic silencing via promoter hypermethylation, all of which contribute to tumor dissemination and metastasis [28]. Promoter hypermethylation of *CDH1* gene represents a key epigenetic mechanism leading to silencing of E-cadherin protein expression and contributing to the pathogenesis of ILC [33].

## 4. Tumor Dormancy in ILCs

Tumor metastasis and dormancy are critical events in tumorigenesis. Metastatic potential refers to the ability of malignant cells, including circulating tumor cells, to detach from the primary tumor and disseminate through lymphovascular channels to distant sites [34]. Following dissemination, a subset of residual malignant cells may enter a quiescent, non-proliferative state known as tumor dormancy. These dormant cells can persist for prolonged periods and subsequently undergo reactivation, leading to metastatic growth at distant sites [35].

The latent appearance of metastatic disease years or even decades after apparent clinical cure of the primary malignancy has been attributed to metastatic tumor dormancy. Dormant cells may include cancer stem cells, a small population of quiescent, non-proliferating, and relatively chemoresistant cells capable of reconstituting the tumor at a later stage. In this state, patients may harbor circulating or disseminated tumor cells without clinically detectable disease [36]. Dormancy is maintained through multiple mechanisms, including tumor microenvironmental factors, cytokine signaling, angiogenic regulation, and metastasis suppressor genes. While many dormant cells may never become clinically apparent, reactivation can result in metastatic growth capable of overcoming growth constraints and therapeutic pressures [36,37].

### 4.1. Dormancy–Reactivation Model

Patients with ILC exhibit higher numbers of disseminated tumor cells (DTCs) than patients with IDC [38], suggesting that dormant DTCs may contribute to the characteristic late recurrences observed in ILC. Rima et al. established an experimental model to investigate dormancy in ILC cell lines and demonstrated that tumor dormancy is a multifaceted process centered on p27Kip1 signaling [39]. Using two-dimensional protein micropatterns, the authors showed that the dormancy response of tamoxifen-resistant MB134-T cells depended on the surrounding protein microenvironment and was associated with a mesenchymal-like phenotype and filopodial formation [38,39].

We propose that ILC cells metastatic to the uterine cervix follow a dormancy–reactivation model. This model is influenced by both the unique cervical microenvironment and the loss of E-cadherin. The uterine cervical stroma contains homeostatic signaling factors that suppress cellular proliferation [40]. It is enriched in anti-angiogenic and dormancy-inducing proteins, particularly thrombospondin-1 (TSP-1), which may maintain DTCs in a quiescent state through stabilization of a high p38 MAPK-to-ERK signaling ratio [41,42,43,44]. As long as p38 activity predominates over ERK signaling, ILC cells may remain viable but non-proliferative [40,43,45]. In addition, the cervical stroma is enriched in angiostatin and may restrict angiogenic activation, thereby limiting the transition of micrometastatic ILC deposits to overt metastatic growth. Resident immune populations, including NK cells, CD8+ cytotoxic T lymphocytes, and antigen-presenting cells, may further contribute to immune-mediated dormancy by preferentially eliminating actively proliferating neoplastic cells while sparing dormant cells [40,45,46,47,48,49].

### 4.2. The Uterine Cervical Stromal and Anti-Angiogenic Factors

We speculate that the uterine cervical stromal microenvironment contains dormancy-inducing proteins that suppress cellular proliferation [40]. Among these, TSP-1 is a potent endogenous inhibitor of angiogenesis. We propose that TSP-1 is expressed both on ILC cell membranes and within the extracellular matrix-rich cervical microenvironment. Ultrastructural studies have demonstrated strong plasma membrane localization of TSP-1 in ILC cells [42], accompanied by expression of integrin subunits αv and α1. The anti-adhesive properties of TSP-1, together with E-cadherin loss, may facilitate the characteristic single-cell invasive pattern of ILC [42]. Neutrophil-mediated degradation of TSP-1 has also been implicated in escape from dormancy in breast cancer [43].

TSP-1 may maintain dormancy by promoting a high p38/ERK signaling ratio [41,42,43,44]. Under these conditions, ILC cells remain viable yet non-proliferative, with p38 signaling contributing to prolonged G0-phase arrest [40,43,45]. Angiostatin, an endogenous anti-angiogenic factor generated through proteolytic processing within the extracellular matrix [50], may further limit angiogenesis within the cervical stroma. Consequently, micrometastatic ILC cells may persist as dormant single cells or small cellular cords rather than progressing to active metastatic growth [40,50].

### 4.3. The Uterine Cervical Stromal and Immune Cells

The cervical stroma contains resident CD8+ cytotoxic T lymphocytes, NK cells, macrophages, dendritic cells, and other antigen-presenting cells [46,47,48]. We propose that these immune populations contribute to immune-mediated dormancy by selectively eliminating proliferating neoplastic cells while sparing dormant ILC cells [40,45,49]. Multiple studies support the presence of these immune cell populations within normal cervical tissue, including tissue-resident memory CD8+ T-cell subsets, granzyme-B-positive NK-like cells, and macrophage populations capable of antigen presentation and MHC II upregulation under inflammatory conditions [46,47,51]. Although these mechanisms are established in other solid tumors, their specific role in cervical ILC metastasis remains speculative and requires direct experimental validation.

### 4.4. The Uterine Cervical Stromal and Anoikis

Anoikis is a specialized form of detachment-induced apoptosis that functions as an important homeostatic mechanism preventing oncogenic transformation [52,53]. Under physiological conditions, the loss of E-cadherin contributes to the activation of anoikis. We propose that metastatic ILC cells evade this safeguard through acquisition of anoikis resistance [52,53]. In ILC, E-cadherin loss is associated with two major consequences: disruption of intercellular adhesion and acquisition of an anoikis-resistant phenotype [41,54]. The loss of adhesion facilitates dissemination to distant sites, whereas anoikis resistance permits survival within the cervical microenvironment despite detachment from the native extracellular matrix [41,55,56].

The dense fibromuscular cervical stroma may represent a hostile metastatic niche. We propose that limited tissue remodeling prevents the β1-integrin clustering required for activation of downstream FAK/Src/ERK signaling pathways. The resulting absence of proliferative stimuli may constrain metastatic ILC cells to a metabolically quiescent G0/G1 state rather than permitting active oncogenic growth [57,58,59]. Dormancy may be further maintained through p27Kip1-mediated cell cycle arrest. Disseminated ILC cells may therefore persist within the cervical stroma until secondary genetic or epigenetic alterations trigger reactivation and metastatic progression [60,61]. These proposed mechanisms remain speculative and require validation in dedicated cervical ILC models.

## 5. Management of Metastatic ILCs to the Uterine Cervix

As no standardized guidelines exist, management of cervical metastasis from breast ILC requires a tailored approach. Our analysis revealed several treatment strategies. For asymptomatic incidental findings, management may involve observation with serial imaging and optimization of systemic endocrine therapy, with local intervention reserved for the development of symptoms [62]. Hysterectomy with bilateral salpingo-oophorectomy (hormonal ablation) was performed in patients with isolated cervical disease or abnormal vaginal bleeding [21,63].

**Figure 3:**
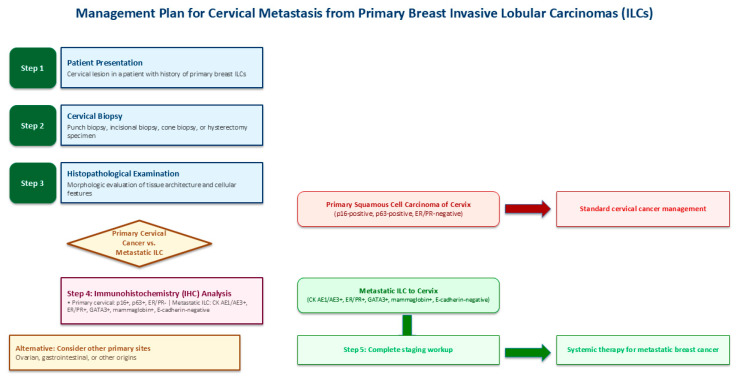
Proposed management and diagnostic algorithm for cervical metastasis from primary invasive lobular carcinoma. The flowchart outlines a systematic 5-step approach for clinicians when managing a patient with a cervical lesion and a history of primary breast ILC. The process begins with clinical identification and tissue biopsy (Steps 1–2), followed by detailed histopathology and specific immunohistochemical (IHC) profiling (Steps 3–4). The algorithm emphasizes the critical role of IHC in differentiating metastatic ILC (CKAE1/AE3+, ER/PR+, GATA3+, mammaglobin+, and E-cadherin−) from primary cervical carcinoma (p16+, p63+, ER/PR−) or other metastatic patterns from ovarian or gastrointestinal origins (Step 5). The final stages involve confirming the diagnosis of metastatic ILC, clinical staging, and implementing a tailored management plan (Step 5).

Recent cases (2024–2026) increasingly incorporated CDK4/6 inhibitors (ribociclib, abemaciclib) in combination with endocrine therapy, reflecting evolving treatment paradigms for metastatic hormone receptor-positive breast cancer. One patient treated with multimodal therapy including surgery, hormonal therapy, and chemotherapy was reported alive at 7-year follow-up. Systemic therapy most commonly involved modification of endocrine regimens [21,64,65]. A summary of the management plan for metastatic mammary ILCs to the uterine cervix is shown in Figure 3.

Endocrine therapeutic agents included aromatase inhibitors (anastrozole, letrozole, exemestane), selective estrogen receptor modulators (tamoxifen), and selective estrogen receptor degraders (fulvestrant). Chemotherapy was administered in some cases typically using regimens containing 5-fluorouracil, epirubicin, cyclophosphamide, docetaxel, or other agents.

Radiotherapy was primarily used for palliative control of symptomatic vaginal bleeding [21,25,63]. In some cases, a combined approach consisting of surgery followed by adjuvant therapy was utilized [2]. Combination therapy included surgery plus endocrine therapy or triple therapy combining surgery, endocrine therapy, and chemotherapy, both representing the most frequent combination. This multimodal approach addresses both local disease control and systemic hormone-dependent tumor growth. The predominance of surgical and endocrine interventions reflects the hormone-sensitive nature of these metastases and the importance of local control for symptom management.

In cases of widespread metastatic disease, management focused on palliative symptom control, including local radiotherapy for bleeding, alongside optimization of systemic therapy for overall disease control [24,66]. A summary of the management plan for metastatic mammary ILCs to the uterine cervix is shown in Figure 3.

## 6. Prognostic Factors

The prognosis for patients with ILC metastasis to the uterine cervix is generally poor, and hysterectomy does not appear to improve outcomes [21,63]. Favorable prognostic indicators include a long disease-free interval of more than three years, isolated cervical metastasis, strong hormone receptor positivity, and good patient performance status [22,23]. Conversely, poor prognostic factors include synchronous presentation of metastasis with the primary breast cancer, the presence of multiple metastatic sites, hormone receptor-negative disease, and poor performance status [67,68].

## 7. Specific Aims of the Review

To the best of our knowledge, some heterogeneous case reports of ILCs metastatic to the uterine cervix have been reported in the English literature [21,22,23,26,64,68,69]. However, comprehensive synthesis of the clinicopathologic features and underlying molecular mechanisms of this metastatic pattern is still lacking. Therefore, our understanding of these lesions remains rudimentary. We conducted this study to improve this understanding by translating heterogeneous clinicopathologic findings from case reports into clear clinicopathologic concepts for practicing gynecologists and pathologists.

## 8. Materials and Methods

### 8.1. Protocol

The authors screened the English language literature and identified limited case reports directly related to this specific topic, making a systematic review impractical. Moreover, these studies were heterogeneous, which precluded performing a meta-analysis. Accordingly, we conducted a narrative review. This approach was more appropriate for synthesizing limited and heterogeneous evidence. This narrative review was conducted according to the SANRA framework [70], the established quality standard for narrative reviews in medical literature.

### 8.2. Search Process and Information Source

This study did not involve any interaction with patients or access to medical records. Accordingly, institutional review board approval was not required. Following the SANRA framework [70], the authors searched PubMed (NLM) database up to March 2026 using combinations of Medical Subject Headings (MeSH) and free-text terms including (“invasive” OR “lobular carcinoma” OR “lobular” OR “carcinoma”) AND (“breast” OR “cervix” OR “mammary”) AND (“cervix uteri” OR “metastasis”) OR (“Primary” OR “secondaries”).

A total of 102 records were identified. Titles and abstracts were screened, and full texts of relevant articles were reviewed. Eligible studies included peer-reviewed, full-length case reports or case series published in the English language. Meeting abstracts, reviews, editorials, and non-English articles were excluded. The rationales behind using PubMed as the sole search engine are: (i) PubMed has a comprehensive indexing of English language case reports in oncology journals, and (ii) given the extreme rarity of this condition (only 29 cases over 67 years), expanding to additional databases would not substantially alter the case pool while significantly increasing screening burden.

### 8.3. Selection Process and Inclusion Criteria

The authors initially screened eligible case reports based on their titles, abstracts, language, and publication dates. Full texts were then examined to determine eligibility for inclusion. The authors independently reviewed and analyzed all eligible studies. The inclusion criteria were: (i) case reports and case series published in the English language containing the aforementioned keywords, (ii) human studies describing cervical metastasis from histologically confirmed breast ILC, (iii) availability of clinical data including patient age, clinical presentation, interval to metastasis, immunohistochemical profile, treatment, or outcome, (iv) prior history of immunohistochemically documented primary mammary ILC, and (v) studies with immunohistochemical confirmation of metastatic ILC.

### 8.4. Exclusion Criteria

The exclusion criteria included: (i) studies not published in the English language literature, (ii) studies lacking evidence of preceding ILC prior to the diagnosis of metastatic ILC to the uterine cervix, (iii) studies lacking immunohistochemical confirmation of metastatic ILC to the uterine cervix, and (iv) articles that were not peer-reviewed. Of the 102 studies identified, 73 were excluded for being non-English, review articles, or not reporting primary case data on cervical metastasis from breast ILC. Peer-reviewed original case reports and case series lacking geographical data were excluded. The final analysis included 29 articles spanning 1959 to March 2026, reporting individual patient cases.

### 8.5. Methodological Quality Assessment

To evaluate the structural and methodological strength of the literature comprising the database, each of the 26 source publications was subjected to an independent study-by-study quality assessment using the *Scale for the Assessment of Narrative Review Articles* (SANRA) framework. The SANRA scores were calculated across six fundamental criteria—rationale, objectives, the literature search description, referencing, scientific reasoning, and data presentation—using a standardized 0-to-2-point scale (maximal score = 12). Studies achieving a SANRA score equal or more than 10 were categorized as high-tier literature reviews, while standalone case reports lacking independent search strategy details were categorized as supportive metadata providers.

### 8.6. Synthesis and Analysis of Data

This review was designed and conducted as a narrative synthesis following established quality guidelines for narrative reviews, specifically the SANRA (Scale for the Assessment of Narrative Review Articles) framework. Data synthesis followed narrative review methodology per SANRA criteria, with descriptive statistics and qualitative thematic analysis of case characteristics. All eligible studies were independently screened, reviewed, and interpreted. The authors independently extracted and collected clinicopathologic data, including patient age, symptoms, interval to metastasis, and diagnostic findings. Histological and immunohistochemical findings were also extracted and synthesized.

### 8.7. Statistical Analysis of the Data

Statistical analysis was performed using IBM-SPSS 21.0. Extracted data fields included patient country of origin, continent, age at presentation, key clinical symptoms, immunohistochemistry (IHC) profile, clinical management pathways, and the specific disease-free interval (latency). For chronological tracking, synchronous metastases (cervical involvement discovered during the initial staging or baseline workup of the primary breast tumor) were mathematically defined as an interval of 0.0 months/years. Continuous demographic variables (age and latency) were summarized utilizing medians, means, and full ranges.

The data were collected, organized into tables, and synthesized to analyze clinicopathologic characteristics of ILC metastatic to the uterine cervix. A summary of these findings is presented in Table 1, Table 2 and Table 3.

## 9. Results

### 9.1. PubMed Literature Analysis

A total of 102 articles were identified from the English language literature, over a span of almost 67-year period (1959–2026). Seventy-three studies were excluded due to: (i) lack of supporting immunohistological findings and (ii) lack of full-text availability in English. The remaining 29 case reports and case series on metastatic mammary ILCs were included in this narrative review and are detailed in Table 1.

### 9.2. Clinical Characteristics

The mean age of patients was 56.8 ± 2.0 years (range, 32–78 years; n = 26). The median age was 56.5 years. The age distribution of patients is shown in Table 1 and Table 2. The majority of patients (n = 12, 41.4%) were in the 50–59-year age group. Five patients (17.2%) were younger than 50 years, five (17.2%) were 60–69 years, and three (10.3%) were 70 years or older. A summary of these findings is shown in Table 1 and Table 2.

The cases have been reported from 14 different countries, spanning Asia (Japan, China, India, Korea, Taiwan, Singapore, Kuwait), Europe (Italy, UK, Turkey, Germany, Serbia, Hungary), North America (the USA), South America (Brazil), and Africa (Egypt). Geographic distribution analysis revealed that nearly half of these cases originated in Asia (n = 14, 48.3%), followed by Europe (n = 9, 31.0%) and North America (n = 4, 13.8%). On a national level, Japan reported the highest frequency of cases (n = 5, 17.2%), followed by the USA (n = 4, 13.8%) and Italy (n = 3, 10.3%). Minor regional representations were reported in Taiwan, India, the UK, and Hungary (n = 2, 6.9% each), while single cases (n = 1, 3.4% each) were reported across China, Korea, Turkey, Kuwait, Singapore, Germany, Serbia, Egypt, and Brazil.

Across the global cohort, the overall median age at presentation was 57.0 years (mean: 56.8 years; range: 32–78 years). Stratification by geographic region demonstrated relatively similar age profiles among different continents. The median age at presentation was 56.0 years in Asia (range: 40–74 years), 57.5 years in North America (range: 50–65 years), and 58.0 years in Europe (range: 32–78 years). Single case reports from Africa and South America documented presentation ages of 59 and 57 years, respectively. These findings indicate that while cervical metastasis from ILCs remains an exceptionally rare occurrence, it consistently presents during the fifth to sixth decades of life, independent of geographic origin. A summary of these findings is shown in Table 3.

### 9.3. Clinical Presentations

Abnormal irregular vaginal bleeding was the most common presenting symptom, occurring in 19 patients (65.5%). This included menorrhagia, postmenopausal bleeding, and abnormal vaginal bleeding of varying duration. Four patients (13.8%) were asymptomatic, with metastases discovered incidentally during routine gynecological examination or imaging. Pelvic or abdominal pain and mass symptoms were present in two patients (6.9%). A summary of these findings is shown in Table 1 and Table 2.

### 9.4. Interval from Primary Diagnosis

The temporal relationship between the primary ILC diagnosis and cervical metastasis varied considerably (Table 1). Synchronous presentation was observed in nine patients (31.0%). Among the 18 metachronous cases with available data, the mean interval was 55.6 ± 8.2 months (median, 45.5 months; range, 23–128 months), corresponding to approximately 4.6 ± 0.7 years. The majority of metachronous cases (n = 12, 41.4% of total) presented 2–5 years after the primary diagnosis. Three patients (10.3%) developed cervical metastasis within 2 years, and three (10.3%) more than 5 years after initial diagnosis. The longest reported interval was 10 years. A summary of these findings is shown in Table 1.

### 9.5. Diagnostic Procedures

In all cases, the diagnosis was based on prior history of ILC, clinical examination, and pathological examination. Imaging modalities (ultrasound, MRI, PET-CT) often revealed a cervical mass, sometimes mistaken for a leiomyoma [69]. Metastatic ILC may also be misdiagnosed as hematolymphoid malignancy based on imaging studies [26,79]. During local examination, the uterine cervix appeared either suspicious and unhealthy (enlarged and indurated) or entirely unremarkable. The diagnosis of metastatic ILCs to the uterine cervix was established through punch biopsy, cervical conization [24], or hysterectomy [21,25,69,77,80,82]. Histologically, the primary differential diagnoses included primary endocervical adenocarcinoma, endometrial carcinoma, and metastasis from other sites (e.g., gastrointestinal tract) [29] (Table 2).

### 9.6. Immunohistochemical Profile

The final confirmation of diagnosis relied heavily on immunohistochemistry confirming breast origin (positive tumor cell reactivity for CK7, GATA3, ER, PR, mammaglobin, GCDFP-15; negative for CK20, PAX8) [30,77,80]. A summary of these findings is shown in Figure 2, Table 1 and Table 2.

The immunophenotype was consistent with the expected profile of ILCS (Table 1). Estrogen receptor (ER) positivity was observed in 12 cases (60.0% of those with available data), and progesterone receptor (PR) positivity in 10 cases (50.0%). E-cadherin negativity, characteristic of ILCS, was present in seven cases (35.0%). GCDFP-15 positivity, confirming breast origin, was seen in eight cases (40.0%). GATA3 positivity was observed in four cases (20.0%), and human epidermal growth factor receptor 2 (HER2) positivity in three cases (15.0%). Cytokeratin 7 (CK7) positivity was present in three cases (15.0%). A summary of these findings is shown in Table 2 and Table 3.

### 9.7. Management

Analysis of the 29 cases indicated that treatment approaches varied based on disease extent, hormonal status, and patient factors (Table 1). Combined surgical and systemic therapy was the most common approach, utilized in 10 patients (34.5%). This typically included total abdominal hysterectomy with bilateral salpingo-oophorectomy (TAH + BSO) followed by hormonal therapy, chemotherapy, or targeted agents. Seven patients (24.1%) underwent surgery alone. Systemic therapy without surgery was administered in two patients (6.9%) and palliative or supportive care in two (6.9%). Treatment details were not specified or refused in eight cases (27.6%).

## 10. Discussion

Metastatic involvement of the uterine cervix from ILCs represents an exceptionally rare but clinically significant pattern of disease spread. The available body of evidence on metastatic ILCs to the uterine cervix remains incomplete, as most published studies are limited to case reports, but comprehensive reviews on this subject are still lacking. This thereby limits our understanding of the clinicopathologic features of this entity. This narrative review synthesized reported cases of ILC metastatic to the uterine cervix, focusing on clinical presentation, immunohistochemical profile, and management strategies. Our study revealed several important observations: (i) uterine cervix is resistant to metastatic involvement from other organs, (ii) metastatic ILCs to uterine cervix occurs after a long disease-free interval (mean of 55.6 months) [64,66], (iii) patients are typically middle-aged (mean age 56.8 years) and most frequently present with abnormal vaginal bleeding [22], (iv) diagnosis is challenging due to its mimicry of primary cervical neoplasms [79], (v) accurate diagnosis relies heavily on confirmatory immunohistochemistry [30], (vi) the unique pathogenesis is primarily driven by loss of E-cadherin protein expression in ILCs [28].

### 10.1. Clinical Presentations in Metastatic ILCs to the Uterine Cervix

This investigation indicates that the majority of patients with metastatic ILCs to the uterine cervix were postmenopausal women, consistent with the typical epidemiology of ILC. The relatively narrow standard error of 2.0 years suggests a homogeneous age distribution centered around the sixth decade of life. The relatively long interval between the onset of primary ILCs and the detection of metastatic deposits in the uterine cervix emphasizes the dormant but persistent nature of ILCs and the necessity for long-term surveillance in breast cancer survivors.

The clinical presentations of breast ILCs metastatic to uterine cervix were similar to those of primary cervical tumors [21,22,23,64,68,69]. However, some cases were completely asymptomatic [22,26,68]. On local examination, the findings resembled those of primary cervical cancer [11,25]. The symptoms were highly non-specific, with abnormal vaginal bleeding being the most common presenting symptom, occurring in almost all cases [11,25]. Taken collectively, these findings emphasize the necessity to keep a high index of suspicion when evaluating the uterine cervix in patients with a history of breast ILCs [2,74].

### 10.2. Geographic Patterns of ILCs Metastatic to the Uterine Cervix

The observed geographical patterns indicate that nearly half of the reported cases originated from Asia (48.3%) followed by Western cohorts. This can be attributed both to population genetics and health care infrastructure in different continents. Published case reports of ILCs metastatic to the uterine cervix did not provide data on the underlying genetic architecture of the cohorts. Therefore, establishing an exact molecular explanation for these demographic patterns remains elusive based on current literature. However, it is possible that these geographical patterns reflect ancestry-associated molecular subtype heterogeneity or the presence of population-specific *CDH1* variants. Moreover, geographical patterns are also heavily affected by the efficiency of the regional health care systems. Countries utilizing efficient, multi-system gynecological follow-ups are more likely to detect clinically silent, occult cervical metastases [1,2,3,4]. The extended latency periods (period between the onset of ILCs and the diagnosis of metastasis to the uterine cervix) strongly support molecular theories regarding microenvironmental tumor tissue dormancy, where detached neoplastic ILC cells survive inside the hostile fibromuscular stroma of the uterine cervix for years before undergoing secondary oncogenic reactivation.

### 10.3. Immunohistochemistry in Metastatic ILCs to the Uterine Cervix

The diagnosis of metastatic breast ILCs to the uterine cervix was established in all cases through histological evaluation followed by immunohistochemical confirmation [21,24,69,77,80]. The immunohistochemical profile demonstrated remarkably consistent features across cases of metastatic ILCs to the uterine cervix. Immunohistochemistry was pivotal in identifying metastatic breast ILCs in all cases. This immunohistochemical signature—ER +, PR +, HER2 -, E-cadherin -, GATA3 +, CK7 +, CK20 -, GATA3 +, mammaglobin +, and GCDFP-15 +—forms a diagnostic panel that reliably distinguishes metastatic ILCs from primary cervical carcinoma and other metastatic deposits. Additional panels included organ-specific markers such as PAX8 (urogenital origin) and CDX-2 (gastrointestinal origin) [30,84,85,86].

GATA3 positivity, a breast-specific transcription factor, was observed in all cases where tested, confirming the breast origin of the metastatic deposits. GCDFP-15 (gross cystic disease fluid protein-15), another breast-specific marker, was present in most cases. CK7 positivity, an epithelial marker, was present in all cases, consistent with epithelial malignancies of breast origin. CK20 negativity was observed in all cases where tested. This pattern (CK7 positive, CK20 negative) is characteristic of breast and gynecologic primaries and helps exclude gastrointestinal sources [30,77,80].

Loss of E-cadherin expression was the most diagnostically significant finding, present in all cases where evaluated. This loss of E-cadherin staining is pathognomonic for ILCs and serves as the key distinguishing feature from IDCs and primary cervical malignancies. The consistent loss of this cell adhesion molecule explains the discohesive growth pattern and infiltrative nature of ILCs metastases [32].

The hormone profile status of breast ILCs metastatic to the uterine cervix closely resembled those of primary ILCs. Most cases retained reactivity for hormonal receptors (ER and PR) [2,23,69]. In contrast, human epidermal growth factor receptor 2 (HER2) overexpression was absent in most evaluated metastases [21,22]. The universal ER positivity explains the typical response to endocrine therapy observed in these patients. Similarly, the equally universal PR positivity reinforces the luminal A molecular subtype characteristic of ILC [2,23,69]. Alternatively, the observed HER2 negativity in most cases confirms the predominantly luminal A phenotype of metastatic lobular carcinoma to the uterine cervix [21,22].The consistency of this profile across metastases, even those presenting years after the initial diagnosis, as in the 11-year interval case reported by Gerber et al. [66], confirms the phenotypic stability of breast ILCs during their progression and validates the use of immunohistochemistry for accurate diagnosis in patients with a history of breast cancer. Moreover, this immunoprofile—strong ER/PR positivity and HER2 negativity—can help distinguish metastatic ILC from primary cervical adenocarcinomas, which typically exhibit a different immunoprofile pattern [87,88,89].

Metastatic ILCs to the uterine cervix and primary endometrial carcinoma were both reactive for ER and PR; therefore, these markers were not useful in distinguishing between these tumors. The distinguishing markers in this context include PAX8 (for endometrial origin), gross cystic disease fluid protein 15 (GCDFP-15), mammaglobin, and GATA3 (for mammary origin) [30,84,85]. GATA3 is a transcription factor involved in the development and differentiation of luminal breast cells [90,91]. It is an important immunostain for diagnosis of ILCs metastatic to the uterine cervix. This is because of its high expression rates in ILCs reaching about 100%. Moreover, GATA3 is rarely expressed in endometrial carcinoma, cervical adenocarcinoma or ovarian mucinous carcinoma [30].

### 10.4. Diagnostic Challenges

Diagnostic confusion in metastatic primary ILCs to the uterine cervix was attributed to the rarity of this disease entity and its clinical presentation mimicking primary cervical carcinoma. Histologically, the single-file infiltration pattern of metastatic ILC within the cervical fibromuscular stroma can also be encountered in primary cervical adenocarcinoma. The diagnosis of metastasis from ILCs to the uterine cervix was established through various approaches, with local examination and cervical punch or cone biopsy being the most frequently employed diagnostic methods. This tissue diagnosis was essential not only for confirming metastatic disease but also for distinguishing it from primary cervical malignancies. Physical examination contributed to diagnosis in most cases, typically by detecting an indurated, enlarged, or hard cervical mass that raised clinical suspicion. The diagnostic challenge in these rare cases was markedly compounded by the subtlety of radiological findings. In many cases, imaging studies such as MRI and ultrasound demonstrated only diffuse cervical enlargement without a definite cervical mass [26,68]. The roles of imaging studies (CT, MRI, or PET-CT) included staging, assessment of disease extent, and detection of additional metastatic sites [26,68].

Cancer screening led to incidental detection of some cases of ILCs metastatic to the uterine cervix, particularly through cervical cytology abnormalities or routine postmenopausal bleeding evaluation [24,25,26]. However, cervical Pap smear had low sensitivity for detecting metastatic ILC and for distinguishing it from primary cervical carcinoma [24,25,26]. Taken together, the most common specific and successful diagnostic combination was physical examination plus biopsy, highlighting the importance of thorough pelvic examination in breast cancer survivors with gynecologic symptoms.

### 10.5. Prognosis

The prognosis of ILCs to uterine cervix is almost always dismal. There are no consensus protocols for diagnosis or treatment. Optimal outcomes depend primarily on early diagnosis and the use of multidisciplinary management, including chemotherapy, radiotherapy, and hysterectomy [63,64]. Despite the dismal outcome, some cases achieved complete remission. Others remained disease-free for a long time, exceeding 20 years [92]. Overall, outcomes were generally unfavorable, with a low 5-year survival rate [2].

### 10.6. Treatment and Prognosis

There are no consensus treatment protocols for ILCs metastatic to the uterine cervix [63,64]. Therefore, the management of this rare malignancy required a tailored and multimodal approach based on disease extent, symptoms, and patient factors. The therapeutic regimens included observation with serial imaging, optimization of systemic endocrine therapy [62], surgical resection, and radiation therapy [21,63].

Surgery (total abdominal hysterectomy with bilateral salpingo-oophorectomy or radical hysterectomy) was the most common single therapeutic modality, performed in most cases. Surgical intervention served multiple purposes: local disease control, relief of bleeding symptoms, removal of hormone-producing ovaries (ovarian ablation or castration), and prevention of complications such as profuse vaginal discharge, and obstruction of the cervical canal. This utilization of endocrine therapy reflects the universal ER and PR hormone receptor positivity observed in these tumors.

### 10.7. Strength of the Study

Our current review has several strengths. Importantly, its theme addresses a clinically aggressive malignancy that remains poorly understood. We identified 102 studies in the English language literature published over a span of almost 67-year period (1959–2026), and this extended timeframe ensured a thorough assessment of the reported cases. Our research expands the current literature by comprehensively summarizing the clinicopathologic features of metastatic mammary ILCs to the uterine cervix. This review also highlights the importance of immunohistochemical confirmation and multidisciplinary management for this unusual metastatic pattern.

### 10.8. Limitations of Evidence

The evidence synthesized in this narrative review has some limitations. One important limitation is the restriction of the search strategy to PubMed-indexed articles. Additional limitations include restriction to articles published in the English language. The heterogeneity of the included studies precluded meta-analysis. Furthermore, the limited number of eligible investigations made a systematic review impractical. Consequently, no formal risk-of-bias assessment was conducted.

## 11. Conclusions

Metastasis from primary mammary ILC represents a unique clinicopathologic entity with challenging diagnostic and therapeutic implications. The rarity of such metastases is attributed to the uterine cervical fibromuscular stroma, which may inhibit tumor growth, along with the organ’s small size, limited blood supply, outward-only lymphatic drainage, and minimal exposure to peritoneal fluid [2,63,93]. The main conclusion of this review is the necessity for keeping a high index of suspicion when evaluating any uterine cervical abnormality in a patient with a history of cancer [2,74]. The diagnostic approach should include a complete clinical history, thorough clinical and radiological examination, cervical cytology, punch biopsy, and immunobiological evaluation, particularly the use of GATA3, to confirm breast origin [2,74].

Moreover, this review underscores the critical need for gynecologists and pathologists to maintain a high index of suspicion for metastatic disease in breast cancer patients presenting with new gynecologic symptoms, even many years after initial treatment. Long-term, multidisciplinary follow-up incorporating gynecological assessment is essential for the timely detection and management of such atypical metastases.

## 12. Future Research Directions

Our study raises several unanswered questions: which patients with primary breast ILC should undergo follow-up, what is the appropriate interval for follow-up, what are the optimal cost-effective surveillance tools for patients with breast ILCs, and whether artificial intelligence can be used to develop risk prediction models that would help identify patients with a history of breast ILC who are at high risk for developing metastatic disease. It is tempting for future studies to examine if this approach can facilitate timely follow-up and referral. This would be particularly beneficial in busy outpatient clinics and hospitals. Future systematic reviews on this topic should incorporate other search engines such as Embase, Cochrane, and Scopus for completeness. The substantial geographic diversity of the reported case reflects the ubiquitous nature of cervical metastasis from ILCs. It also emphasizes the critical need for large, multi-center, population-based studies that incorporate comprehensive genomic and ancestry data to examine the role of genetic background in this rare metastatic event.

## Figures and Tables

**Table 1:** Reported cases of metastatic invasive lobular carcinoma to the uterine cervix. TAH: total abdominal hysterectomy. BSO: bilateral salpingo-oophorectomy.

Case/ Country	Age (Years)	Clinical Presentations	Interval from Primary ILC Diagnosis To Cervical Metastasis	Immunohistochemistry	Management	Reference
1 Italy	60	Abnormal vaginal bleeding, acute renal failure, enlarged cervix with obliterated fornices.	10 years	ER+, PR+, HER2−, E-cadherin−, GATA3+, CK7+, CDX2−, CK20−.	Ribociclib for renal failure; CDK4/6 inhibitor and endocrine therapy.	[7]
2 China	40	Asymptomatic. The metastases were discovered during routine gynecological examination.	7.5 years	ER +, PR +, HER2 −, GATA3 +, GCDFP-15 +, E-cadherin−.	TAH + BSO and combination of fulvestrant and abemaciclib.	[71]
3 USA	65	Abnormal vaginal bleeding.	2 years	Pancytokeratin (AE1/AE3) +, GATA3+	TAH + BSO CDK-4/6 inhibitor (ribociclib) and radiotherapy.	[72]
4 Taiwan	57	Postmenopausal bleeding, new breast mass.	2 years	CK7+, ER+, GATA-3+ and CD10−, CK20−, and CDX2−.	TAH + BSO and CDK4/6 inhibitor (ribociclib).	[23]
5 Japan	66	Abnormal vaginal bleeding and enlarged cervix.	23 months	ER+, PR+, HER2+, E-cadherin−, GCDFP-15+	TAH + BSO + peritoneal biopsy + hormonal therapy.	[73]
6 Taiwan	57	Abnormal uterine bleeding.	30 months	CK7+, ER+, and GATA-3+.	TAH + BSO, fulvestrant and CDK4/6 inhibitor (ribociclib).	[23]
7 USA	53	Abnormal vaginal bleeding.	Synchronous	Tumor cells detected during Pap smears. Pancytokeratin (AE1/AE3) + and CK 7+.	Not specified.	[74]
8 Egypt	59	Abnormal vaginal bleeding and pelvic pain.	Synchronous	ER−, PR−, HER2−, CK7+, GATA3+.	TAH + BSO + palliative chemotherapy.	[75]
9 Brazil	57	Abnormal vaginal bleeding and abdominal discomfort.	>3 years	ER+, PR+, HER2+, E-cadherin−, BRST2−.	Anastrozole followed by TAH + BSO.	[22]
10 UK	32	Abnormal vaginal bleeding.	Sometime later	Endometrial and cervical polyps. Tumor cells were CK7+, GCDFP15+ ER−, PR−.	Biopsies from the polyps.	[3]
11 Korea	46	Menorrhagia, cervical mass resembling submucosal leiomyoma.	2 years	ER+, PR+, GCDFP-15+, CK+.	TAH + BSO, second-line chemotherapy.	[69]
12 USA	62	A Pap smear demonstrated simultaneous metastasis of the cervix and endometrium.	Synchronous	ER+, PR+, pancytokeratin (AE1/AE3) +, GATA3, and PAX8 −.	The patient refused further management.	[76]
13 Japan	58	Abnormal uterine bleeding, endometrial polyp, and leiomyoma.	9 years	ER +, PR +, HER2 −, E-Cadherin−.	TAH + BSO, colectomy.	[77]
14 India	49	Menorrhagia for 6 months.	Synchronous	ER+, PR+, HER2−.	TAH + BSO followed by palliative chemotherapy.	[63]
15 India	49	Abnormal vaginal bleeding, abdominal pain, and hard cervical mass.	4 years	ER+, PR+, HER2−, E-cadherin−, CK+.	Chemotherapy with carboplatin and gemcitabine.	[63]
16 Japan	62	Abnormal vaginal bleeding.	7 years	ER+, pancytokeratin (AE1/AE3) +, 7+, GCDFP-15+, and mammaglobin +, and E-cadherin−.	TAH + BSO and exemestane hormonal therapy.	[78]
17 Japan	52	Large cervical tumor (initially diagnosed as leiomyoma).	Synchronous	ER+, PR+, HER2−.	TAH + BSO, mastectomy, hormone therapy, and chemotherapy; alive at 7-year follow-up.	[79]
18 Italy	78	Enlarged cervix on routine examination.	Synchronous	CK7+, CK903 (34E12)+, CK20−, E-cadherin−, ER−, and PR−.	Multisystemic medical treatment including radiotherapy.	[68]
19 Turkey	56	Vaginal bleeding on anastrozole	3 years	Pancytokeratin (AE1/AE3) +, GCDFP-15+.	Adjuvant anastrozole, TAH + BSO.	[21]
20 Serbia	56	Uterine malignancy	52 months	CEA+, GCDFP-15+	TAH + BSO.	[80]
21 Kuwait	50	Postmenopausal bleeding, pelvic pain, enlarged firm indurated cervix; metastatic cells detected on Pap smear.	Synchronous	Punch cervical biopsy performed.	Not specified.	[81]
22 UK	78	Postmenopausal bleeding on tamoxifen	Not specified	CK7+, EMA+, CK 20−, CA 125−, CEA− melanoma markers −, lymphocyte markers −.	Hysterectomy.	[65]
23 Hungary	Not specified	Asymptomatic (cytology finding)	43 months	Not specified.	Not specified.	[62]
24 Japan	Not specified	Abnormal genital bleeding	10 years, 8 months	CA15-3+, ER−, HER2/neu −.	Hysterectomy + chemoendocrine treatment.	[64]
25 Hungary	Not specified	Asymptomatic (ultrasound finding)	53 months	Not specified.	Not specified.	[62]
26 USA	50	Abdominal distension, abnormal Pap smear.	Synchronous	ER+, PR+.	TAH + BSO.	[26]
27 Singapore	74	Normal-appearing cervix, firm and indurated on palpation, abnormal cytology.	4 years	Pancytokeratin (AE1/AE3) +, desmin−, and CD34−.	Colposcopy and biopsy. No further treatment mentioned by the authors.	[25]
28 Italy	Not specified	Abnormal bleeding on tamoxifen, cervical polyp.	2 years into therapy	Not specified.	TAH + BSO.	[82]
29 Germany	55	Abdominal pain and cervical mass.	Synchronous	Not specified.	Excision of cervical tumor.	[83]

**Abbreviations:** TAH: total abdominal hysterectomy, BSO: bilateral salpingo-oophorectomy, ILC: invasive lobular carcinoma, ER: estrogen receptor, PR: progesterone receptor, HER2/HER2/neu: human epidermal growth factor receptor 2, CK: cytokeratin, CK7: cytokeratin 7, CK20: cytokeratin 20, CK903 (34E12): cytokeratin 903 (high-molecular-weight cytokeratin), AE1/AE3: anti-pancytokeratin antibody clone (used to detect epithelial cells), GATA3/GATA-3: GATA binding protein, GCDFP15: gross cystic disease fluid protein, BRST2: breast carcinoma-associated antigen (often synonymous with GCDFP-15), CDX2: caudal-type homeobox transcription factor 2, CD10: cluster of differentiation 10, CD34: cluster of differentiation 34, PAX8: paired box gene 8, EMA: epithelial membrane antigen, CEA: carcinoembryonic antigen, CA 125: cancer antigen 125, CA15-3: cancer antigen 15-3, CDK4/6/CDK-4/6: cyclin-dependent kinase 4 and 6 (inhibitor therapy), PAP/Pap: Papanicolaou test (Pap smear), UK: United Kingdom and USA: United States of America.

**Table 2:** The clinicopathologic features of the reported cases of metastatic invasive lobular carcinoma (ILC) to the uterine cervix.

Category	Characteristic	n	%
**Demographics**	Age < 50 years	5	17.2
	Age 50–59 years	12	41.4
	Age 60–69 years	5	17.2
	Age ≥ 70 years	3	10.3
	Age not specified	4	13.8
**Clinical presentation**	Abnormal vaginal/uterine bleeding	19	65.5
	Pelvic/abdominal pain or mass	2	6.9
	Asymptomatic (routine examination)	4	13.8
	Other/not specified	4	13.8
**Interval from primary** **ILC diagnosis**	Synchronous	9	31.0
	<2 years	3	10.3
	2–5 years	12	41.4
	>5 years	3	10.3
	Not specified	2	6.9
**Immunohistochemistry** **(n = 20 with data)**	ER-positive	12	60.0
	PR-positive	10	50.0
	HER2-positive	3	15.0
	E-cadherin-negative	7	35.0
	GATA3-positive	4	20.0
	GCDFP-15-positive	8	40.0
	CK7-positive	3	15.0
	Not specified/available	9	—
**Management**	Surgery (TAH + BSO) alone	7	24.1
	Surgery + systemic therapy	10	34.5
	Systemic therapy only	2	6.9
	Palliative/supportive care	2	6.9
	Not specified/refused	8	27.6

**Abbreviations:** TAH, total abdominal hysterectomy; BSO, bilateral salpingo-oophorectomy; ER, estrogen receptor; PR, progesterone receptor; HER2, human epidermal growth factor receptor 2; CK, cytokeratin; GCDFP-15, gross cystic disease fluid protein-15; ILC, invasive lobular carcinoma.

**Table 3:** Geographic patterns of invasive lobular carcinoma metastatic to the uterine cervix.

Continent	Country	Case Numbers	Total Count	Percentage (%)
Asia	Japan	5, 13, 16, 17, 24	5	17.2%
	Taiwan	4, 6	2	6.9%
	India	14, 15	2	6.9%
	China	2	1	3.4%
	Korea	11	1	3.4%
	Turkey	19	1	3.4%
	Kuwait	21	1	3.4%
	Singapore	27	1	3.4%
Asia total count	—	—	14	48.3%
Europe	Italy	1, 18, 28	3	10.3%
	UK	10, 22	2	6.9%
	Hungary	23, 25	2	6.9%
	Serbia	20	1	3.4%
	Germany	29	1	3.4%
Europe total count	—	—	9	31.0%
North America	USA	3, 7, 12, 26	4	13.8%
North America Total	—	—	4	13.8%
Africa	Egypt	8	1	3.4%
Africa total count	—	—	1	3.4%
South America	Brazil	9	1	3.4%
South America total count	—	—	1	3.4%
Global total count	—	—	29	100%

“—” from the entire table.

## Data Availability

All the data are included in the manuscript.

## Abbreviations

AE1/AE3, anti-pan cytokeratin antibody cocktail; AWD, alive with disease; BSO, bilateral salpingo-oophorectomy; BRST2, breast carcinoma-associated antigen; CA 125, cancer antigen 125; CA15-3, cancer antigen 15-3; CD10, cluster of differentiation 10; CD34, cluster of differentiation 34; CDK4/6, cyclin-dependent kinase 4 and 6; CEA, carcinoembryonic antigen; CDX2, caudal-type homeobox transcription factor 2; CK, cytokeratin; CK7, cytokeratin 7; CK20, cytokeratin 20; CK903 (34E12), cytokeratin 903; CKAE1/AE3, cytokeratin AE1/AE3; CKMNF116, cytokeratin MNF116; DOD, dead of disease; EMA, epithelial membrane antigen; ER, estrogen receptor; GATA3, GATA-binding protein 3; GCDFP-15, gross cystic disease fluid protein 15; H&E, hematoxylin and eosin; HER2, human epidermal growth factor receptor 2; IDC, invasive ductal carcinoma; ILC, invasive lobular carcinoma; MRI, magnetic resonance imaging; NED, no evidence of disease; NLM, National Library of Medicine; NS, not specified; PAP, Papanicolaou test; PAX8, paired box gene 8; PET-CT, positron emission tomography–computed tomography; PR, progesterone receptor; SANRA, Scale for the Assessment of Narrative Review Articles; SD, standard deviation; SEM, standard error of the mean; TAH, total abdominal hysterectomy; TAH ± BSO, total abdominal hysterectomy with or without bilateral salpingo-oophorectomy; UK, United Kingdom; USA, United States of America.
